# Health Care Expenditures for Black and White US Adults Living Under Similar Conditions

**DOI:** 10.1001/jamahealthforum.2023.3798

**Published:** 2023-11-03

**Authors:** Lorraine T. Dean, Yuehan Zhang, Rachael R. McCleary, Rahel Dawit, Roland J. Thorpe, Darrell Gaskin

**Affiliations:** 1 Department of Epidemiology, Johns Hopkins Bloomberg School of Public Health, Baltimore, Maryland; 2 Johns Hopkins Center for Health Disparities Solutions, Baltimore, Maryland; 3 Analysis Group Inc, New York, New York; 4 Department of Health Policy & Management, Johns Hopkins Bloomberg School of Public Health, Baltimore, Maryland; 5 Johns Hopkins Alzheimer’s Disease Resource Center for Minority Aging Research, Johns Hopkins Bloomberg School of Public Health, Baltimore, Maryland; 6 Department of Health, Behavior and Society, Johns Hopkins Bloomberg School of Public Health, Baltimore, Maryland

## Abstract

**Importance:**

Evidence suggests that racial disparities in health outcomes disappear or diminish when Black and White adults in the US live under comparable living conditions; however, whether racial disparities in health care expenditures concomitantly disappear or diminish is unknown.

**Objective:**

To examine whether disparities in health care expenditures are minimized when Black and White US adults live in similar areas of racial composition and economic condition.

**Design, Setting, and Participants:**

This cross-sectional study used a nationally representative sample of 7062 non-Hispanic Black or White adults who live in 2238 of 2275 US census tracts with a 5% or greater Black population and who participated in the Medical Expenditure Panel Study (MEPS) in 2016. Differences in total health care expenditures and 6 specific categories of health care expenditures were assessed. Two-part regression models compared expenditures between Black and White adults living in the same Index of Concentration at the Extremes (ICE) quintile, a measure of racialized economic segregation. Estimated dollar amount differences in expenditures were calculated. All analyses were weighted to account for the complex sampling design of the MEPS. Data analysis was performed from December 1, 2019, to August 7, 2023.

**Exposure:**

Self-reported non-Hispanic Black or non-Hispanic White race.

**Main Outcomes and Measures:**

Presence and amount of patient out-of-pocket and insurance payments for annual total health care expenditures; office-based, outpatient, emergency department, inpatient hospital, or dental visits; and prescription medicines. ICE quintile 5 (Q5) reflected tracts that were mostly high income with mostly White individuals, whereas Q1 reflected tracts that were mostly low income with mostly Black individuals.

**Results:**

A total of 7062 MEPS respondents (mean [SD] age, 49 [18] years; 33.1% Black and 66.9% White; 56.1% female and 43.9% male) who lived in census tracts with a 5% or greater Black population in 2016 were studied. In Q5, Black adults had 56% reduced odds of having any health care expenditures (odds ratio, 0.44; 95% CI, 0.27-0.71) compared with White adults, at an estimated $2145 less per year, despite similar health status. Among those in Q5 with any expenditures, Black adults spent 30% less on care (cost ratio, 0.70; 95% CI, 0.56-0.86). In Q3 (most racially and economically integrated), differences in total annual health care spending were minimal ($79 annually; 95% CI, −$1187 to $1345).

**Conclusions and Relevance:**

In this cross-sectional study of Black and White adults in the US, health care expenditure disparities diminished or disappeared under conditions of both racial and economic equity and equitable health care access; in areas that were mostly high income and had mostly White residents, Black adults spent substantially less. Results underscore the continuing need to recognize place as a contributor to race-based differences in health care spending.

## Key Points

**Question:**

Are health care expenditures similar among Black and White adults in the US who live under similar economic conditions?

**Findings:**

In this cross-sectional study of a nationally representative sample of 7062 non-Hispanic Black or White US adults, health care spending for Black adults in the US was equal to or less than that of White adults, but only in areas of racial and economic equity and equitable insurance access.

**Meaning:**

Addressing health care expenditure disparities may require understanding how social and economic conditions of patients contribute to differences in expenditures and to equitable insurance access.

## Teaser

This cross-sectional study examines disparities and similarities in health care expenditures among Black and White adults who live under similar economic conditions in the US.

## Introduction

In 2011, the groundbreaking Exploring Health Disparities in Integrated Communities (EHDIC) study found that racial disparities in hypertension, diabetes, obesity among women, and use of health services either disappeared or substantially diminished when comparing a sample of Black and White residents of Baltimore, Maryland, who lived in racially integrated neighborhoods under comparable living conditions.^1^ The results suggested that the social environment may largely drive racial health outcomes in US populations, emphasizing the role of place in understanding health disparities; however, that study left the question of whether, despite equal health outcomes, racial disparities in health expenditures would disappear or diminish.

Even at equal levels of health, racial disparities in health care expenditures could arise due to care that is delayed, not recommended, or avoided because of structural and interpersonal racism^2^ or due to differences in quality of insurance or treatment.^3^ Expenditures may be higher for Black individuals if delays lead to higher health care expenditures when unmet health needs escalate and become urgent and critical or if patients are shifted into more costly health insurance plans. Expenditures could be lower if Black adults are unable to be retained in care due to biases in the health care system^2^ or if care is underused due to differences in which practitioners are in network or due to insurance reimbursement structures.

Given the persistence and size of health disparities between Black and White individuals,^4^ and in follow-up to the EHDIC study,^1^ our analysis intended to answer the question of whether health care expenditure differences are minimized when Black and White individuals live in similar areas in the US. We defined areas that are similar by levels of racial and economic segregation. As an advancement from previous studies that have looked at health care outcomes in 1 city,^1,5,6^ our analysis focused on health care expenditures of Black and White residents in census tracts across the entire US. We hypothesized that there would be no difference in health care expenditures by race when Black and White adults live under similar conditions of economic and racial segregation, in line with previous studies that suggest no difference in health outcomes by race when Black and White people live under similar conditions.^1,5,6^

## Methods

### Data Sources

For this cross-sectional study, we used individual-level demographic, socioeconomic, health status, health care use, and health care expenditure data from the 2016 Medical Expenditure Panel Survey (MEPS), collected by the Agency for Healthcare Research and Quality, which includes a nationally representative sample of the US noninstitutionalized population. MEPS-restricted data were merged with census tract–level area characteristics data from the 2013 to 2017 American Community Survey. The institutional review board at the Johns Hopkins Bloomberg School of Public Health determined that this study did not require institutional review board oversight because the project involved conducting secondary analysis of deidentified data. This study followed the Strengthening the Reporting of Observational Studies in Epidemiology (STROBE) reporting guideline.^7^

### Study Population

The analytic sample included 7062 Black or White MEPS participants aged 21 years or older who live in 2238 of 2275 US census tracts from 47 states where the population is at least 5% Black. We excluded participants with outlier expenditures (total expenditures >$100 000, representing <0.5% of eligible participants) and any participant for whom we could not calculate an Index of Concentration at the Extremes (ICE) value. Another 335 participants were excluded from the analytical sample who did not have positive sampling weight. The eFigure in Supplement 1 presents the flowchart of sample selection. We excluded participants of Hispanic ethnicity and persons of other race groups (American Indian or Alaska Native, Asian, Native Hawaiian or Pacific Islander, and those who reported multiple races). Given the nature and history of segregation of Black and White individuals in the US, as well as the unique needs of more recently immigrated populations, the mechanisms underlying differences in health care expenditures for these other populations may be different and warrant separate analysis.

### Health Care Expenditures

The dependent variables were a binary variable that indicated health care expenditure and a continuous variable for the total amount of health care expenditure on health care services in 2016. In addition, variables were generated for 2016 health care expenditure by category of health service: office-based visits (eg, primary care and imaging tests), outpatient visits (eg, hospital-based care that does not require an overnight stay), emergency department (ED) visits, inpatient hospital stays, prescription medicines, and dental care visits. Expenditures in the MEPS are defined as the sum of direct payments, which include both the out-of-pocket payments and payments made by insurance.

### Race and Race-Income Segregation

The independent variable was MEPS participant race, categorized as non-Hispanic Black or non-Hispanic White (hereafter referred to as Black and White). We excluded participants of Hispanic ethnicity and of other race groups. The analysis was stratified by census tract–level ICE values for race and income, a marker of racialized economic segregation.^8^ The ICE measure was calculated as the difference between the number of White persons in high-income households (annual household income ≥$100 000) and Black persons in low-income households (annual household income <$20 000), divided by the total population with known income in the same census tract.^9^ Quintiles (Q1-Q5) were selected to define strata to be consistent with prior research^9^ using the ICE measure. Quintiles for the ICE measure were computed based on the distribution among census tracts of all MEPS participants aged 21 years or older who lived in census tracts where at least 5% of the population was Black. ICE Q1 had the most population concentrated into the most deprived groups (low income, mostly Black individuals), and ICE Q5 had the most population concentrated into the most privileged groups (high income, mostly White individuals).

### Statistical Analysis

Characteristics and health care expenditures of participants were summarized and compared across ICE quintiles using the Pearson χ^2^ statistic for categorical variables and the Wald test after fitting weighted linear regression for continuous variables. Next, characteristics of Black participants were compared with characteristics of White participants living in ICE Q1 and Q5 (2 groups of census tracts with the most racialized economic segregation) and Q3 (the least racially and economically segregated census tracts).

Two-part models were constructed to model health care expenditures (the overall expenditure and type-specific expenditures) comparing Black participants with White participants living in the same ICE quintile. Next, the incremental expenditures comparing Black participants with White participants (ie, the marginal effects) were estimated based on the combined part 1 and part 2 of the models. Part 1 of the model was a logit model estimating the odds of having any expenditures, yielding odds ratios (ORs) of having any total or type-specific health care expenditures, comparing Black participants with White participants. Part 2 of the model was a generalized linear model with a γ-distribution and log link to model the positive expenditures conditional on having any expenditures, yielding cost ratios (CRs) among persons with positive expenditure, comparing Black participants with White participants. The γ-family distribution was selected for the generalized linear model component based on results of the modified Park test.^10,11^ The 2-part model has been widely used to model health care expenditure because it may account for a large mass at zero (ie, observations with zero expenditures) and skewed distributions.^12^

Two-part models were adjusted for variables hypothesized to be potential confounders: continuous age, sex, educational level, family income as percentage of the poverty line, employment, having any public or private insurance, number of comorbidities,^13^ self-reported health, having a usual source-of-care practitioner, and family having problems paying medical bills in the past 12 months. Census tract–level American Community Survey variables included percentage of population with high school education and percentage of population 65 years or older. Only participants with complete data were included in the regression models (6750 of 7062 [95.6%]).

All analyses were weighted to account for the complex sampling design of the MEPS. A 2-sided *P* < .05 was considered statistically significant. Analyses were conducted using Stata software, version 14.2 (StataCorp). Data analysis was performed from December 1, 2019, to August 7, 2023.

## Results

Of the total 7062 MEPS Black or White respondents who lived in census tracts with a 5% or greater Black population in 2016, 33.1% identified as Black and 66.9% identified as White. As indicated in Table 1, the distributions of age (overall mean [SD], 49 [18] years) and sex (52.6% female and 47.5% male overall) were similar across income-race ICE quintiles; however, most other demographic characteristics of residents in these census tracts varied significantly by income-race ICE quintiles. Higher ICE quintiles (ie, areas with greater White racial and economic privilege) had significantly fewer Black respondents, more people with post–high school education, higher income, less poverty, higher levels of employment and insurance, better mental and physical health, fewer comorbidities, fewer reports of difficulty paying medical bills, and highest access to a usual source of care.

**Table 1.** Characteristics of Study Participants Overall and by Census Tract–Level ICE Quintiles^a^

Characteristic	Overall (N = 7062)	ICE income-race quintiles					*P* value
		1 (Low income, mostly Black people) (n = 1791)	2 (n = 1214)	3 (n = 1201)	4 (n = 1398)	5 (High income, mostly White people) (n = 1458)	
Race and ethnicity, %							
Black	33.1	78.4	47.9	32.6	16.5	13.2	<.001
White	66.9	21.6	52.1	67.4	83.5	86.8	
Age, mean (SD), y	49 (18)	48 (18)	48 (18)	48 (18)	49 (17)	50 (17)	.08
Sex, %							
Male	47.5	45.8	46.4	47.7	47.1	49.1	.40
Female	52.6	54.2	53.6	52.3	52.9	50.9	
Years of education, %							
<12	10.1	17.9	12.7	8.9	9.2	5.6	<.001
12	33.2	41.0	37.2	35.9	33.9	24.3	
>12	56.7	41.0	50.9	55.2	56.9	70.1	
Total family income, median (IQR), $	59 000 (28 447-100 000)	33 226 (15 492-60 576)	50 000 (25 592-82 160)	46 230 (24 927-80 037)	64 000 (35 000-102 134)	86 127 (47 967-130 050)	<.001
Family income level, %							
Poor (<100%)	12.2	23.8	14.8	13.2	8.7	6.4	<.001
Near poor (100%-124%)	3.7	7.8	5.5	4.0	2.3	1.4	
Low (125%-199%)	12.0	17.7	12.6	16.3	1.5	7.1	
Middle (200%-399%)	30.4	32.2	30.6	33.3	33.2	25.1	
High (≥400%)	41.6	18.3	36.5	33.3	45.1	59.9	
Have any employment, %							
No	30.4	39.1	31.9	29.5	27.8	27.1	<.001
Yes	69.4	60.9	68.0	70.5	72.2	72.8	
Insurance status, %							
Any private	70.5	49.8	66.3	67.1	76.9	81.1	<.001
Public only	21.9	37.3	23.5	24.4	17.1	14.7	
Uninsured	7.6	12.9	10.2	8.5	5.9	4.3	
No. of comorbidities, %^b^							
0	26.8	26.7	29.3	27.0	25.1	27.1	.28
1	20.1	20.1	19.2	17.5	21.1	21.1	
2-3	27.6	25.4	25.0	30.7	27.9	28.2	
≥4	25.5	27.8	26.5	24.7	25.9	23.6	
Perceived physical health							
Fair to poor	22.7	32.1	23.6	26.2	22.6	14.5	<.001
Good to excellent	77.4	67.9	76.4	73.8	77.4	85.5	
Perceived mental health							
Fair to poor	14.9	22.4	16.4	15.6	14.5	9.7	<.001
Good to excellent	85.1	77.6	83.6	84.4	85.5	90.3	
Family having problems paying medical bills in the past 12 mo	12.3	14.4	12.3	15.0	13.3	8.5	.007
Have a usual source-of-care practitioner	75.1	69.3	75.6	7.5	78.3	78.4	<.001
Census region							
Northeast	15.4	16.7	13.6	15.8	10.7	19.4	.03
Midwest	18.1	22.2	18.2	17.2	16.7	17.3	
South	59.2	59.6	60.9	54.4	65.2	55.6	
West	7.4	1.4	7.3	12.6	7.4	7.6	
Census tract–level % with high school education, mean (SD)	28.7 (9.9)	35.4 (7.2)	30.5 (7.8)	30.5 (9.4)	29.6 (9.1)	22.0 (9.7)	<.001
Census-tract level % aged ≥65 y, mean (SD)	13.3 (6.0)	12.6 (5.2)	12.5 (5.9)	12.6 (6.5)	14.2 (6.1)	13.7 (5.9)	<.001

Abbreviation: ICE, Index of Concentration at the Extremes.

^a^ Data are presented as weighted percentages unless otherwise indicated.

^b^ Comorbidities include ever diagnoses of high blood pressure, heart disease, stroke, emphysema, chronic bronchitis (during the past 12 months), high cholesterol, cancer, diabetes, joint pain (during the past 12 months), arthritis, or asthma (having an episode during the past 12 months).

As shown in Table 2, across income-race quintiles, there was an increasing trend in the median amount and likelihood of expenditures in total health care, office-based care, prescription drug use, and dental services from Q1 to Q5, with a decreasing gradient in ED expenditures. Mean outpatient and inpatient expenditures showed no clear pattern across the gradient. Across all expenditure categories, the percentage having any expenditure followed similar patterns.

**Table 2.** Distribution of Total and Type-Specific Health Care Expenditures by Census Tract–Level Index of Concentration at the Extremes (ICE) Quintile for Black and White Medical Expenditure Panel Study Participants^a^

Health care expenditures	ICE income-race quintiles				
	1 (Low income, mostly Black people)	2	3	4	5 (High income, mostly White people)
**Total health care expenditures**					
Median (IQR)	881 (16-5036)	1284 (190-4999)	1311 (177-5059)	1651 (320-6304)	1653 (393-5596)
Mean (SD)	5123 (10 665)	5252 (11 244)	5519 (11 377)	5943 (10 938)	5349 (10 393)
Having any expenditures, %	75.8	81.2	83.4	87.3	87.4
**Office-based expenditures**					
Median (IQR)	139 (0-727)	327 (0-926)	299 (0-1204)	365 (42-1365)	493 (78-1612)
Mean (SD)	972 (3091)	1080 (2802)	1253 (3519)	1467 (3636)	1654 (3992)
Having any expenditures, %	60.5	69.8	69.7	76.2	78.7
**Outpatient expenditures** ^b^					
Mean (SD)	392 (2605)	465 (2689)	359 (1897)	472 (2226)	461 (2444)
Having any expenditures, %	14.2	18.9	16.2	18.3	17.1
**Emergency department visit expenditures** ^b^					
Mean (SD)	282 (1119)	254 (926)	284 (988)	253 (991)	152 (748)
Having any expenditures, %	19.6	17.9	17.3	16.3	10.1
**Inpatient expenditures** ^b^					
Mean (SD)	1296 (5855)	1200 (5920)	1520 (6679)	1554 (6467)	1018 (4895)
Having any expenditures, %	9.8	8.1	9.7	9.9	6.7
**Prescription drug expenditures**					
Median (IQR)	49 (0-782)	83 (0-927)	81 (0-866)	186 (0-1178)	121 (0-987)
Mean (SD)	1470 (4221)	1551 (4546)	1445 (4335)	1625 (4932)	1345 (4310)
Having any expenditures, %	61.2	65.6	64.9	72.6	70.0
**Dental care expenditures**					
Median (IQR)	0 (0-0)	0 (0-187)	0 (0-151)	0 (0-162)	0 (0-279)
Mean (SD)	124 (464)	264 (842)	289 (1301)	273 (935)	376 (1179)
Having any expenditures, %	21.8	34.7	34.0	35.7	46.7

^a^ Expenditures (in dollars) for health care utilizations in 2016.

^b^ All of the median (IQR) expenditure estimates were zero dollars for every ICE quintile and therefore are not shown.

A comparison of Black and White respondents suggested that even in areas where Black and White populations live under similar conditions, their landscapes are different (Table 3). In Q1, Black respondents were more likely to be female, have fewer years of education, have lower family income, have higher rates of public insurance or uninsurance, and have better mental health than White respondents. In Q3, Black respondents were more likely to be employed and to have no comorbidities but were otherwise demographically similar to White respondents. In Q5, Black respondents were younger and had lower income, greater exposure to poverty, nearly 3 times the rate of uninsurance, and almost twice the likelihood of having problems paying medical bills. The number of comorbidities was similar for Black and White respondents in Q1 and Q5, although Black respondents were likely to have fewer comorbidities in Q3. Ratings of good physical and mental health were similar for Black and White respondents in Q1, Q3, and Q5, except for a significantly greater percentage of Black (79.5%) compared with White (71.0%) respondents reporting good mental health in Q1 (*P* = .01).

**Table 3.** Characteristics of Black and White Medical Expenditure Panel Study Participants Living in Index of Concentration at the Extremes (ICE) Quintiles 1 (Most Racial and Economic Deprivation), 3 (No Deprivation or Privilege Extremes), and 5 (Most Racial and Economic Privilege)^a^

Characteristic	ICE income-race quintile								
	1 (Low income, mostly Black people)			3 (No deprivation or privilege extremes)			5 (High income, mostly White people)		
	Black respondents (n = 1674)	White respondents (n = 212)	*P* value^b^	Black respondents (n = 659)	White respondents (n = 597)	*P* value^b^	Black respondents (n = 370)	White respondents (n = 1137)	*P* value^b^
Age, mean (SD), y	48 (17)	49 (18)	.77	46 (16)	49 (19)	.06	47 (17)	51 (17)	.004
Sex									
Male	43.9	52.6	.02	46.2	48.5	.41	46.3	49.5	.27
Female	56.1	47.4		53.8	51.5		53.7	50.5	
Years of education, %									
<12	18.9	14.6	.04	11.2	7.8	.16	7.9	5.3	.28
12	43.6	31.7		38.1	34.9		26.4	23.9	
>12	37.6	53.7		50.7	57.3		65.7	70.8	
Family’s total income, median (IQR), $	32 356 (15 000-59 222)	41 964 (16 640-80 495)	.06	48 000 (24 466-84 000)	45 040 (25 000-80 037)	.95	73 066 (36 000-113 561)	90 000 (50 000-131 329)	<.001
Family income level as percentage of the poverty line									
Poverty (<100%)	25.9	16.4	.08	13.8	12.8	.42	9.5	6.0	.004
Near poverty (100%-124%)	7.6	9.0		4.5	3.8		3.3	1.2	
Low (125%-199%)	18.6	14.7		19.4	14.7		12.1	6.4	
Middle (200%-399%)	32.3	31.8		33.4	33.2		26.1	24.9	
High (≥400%)	15.6	28.2		28.9	35.4		49.1	61.6	
Have any employment									
No	38.4	41.6	.51	25.0	31.7	.04	23.3	27.7	.26
Yes	61.6	58.4		75.0	68.3		76.6	72.3	
Insurance status									
Any private	47.3	59.0	.04	65.4	68.0	.50	69.9	82.8	<.001
Public only	39.6	29.1		24.6	24.2		20.7	13.7	
Uninsured	13.2	11.9		10.0	7.8		9.4	3.5	
No. of comorbidities^c^									
0	28.6	20.0	.09	29.3	26.0	.009	33.3	26.2	.23
1	19.5	22.5		22.5	15.1		19.6	21.4	
2-3	25.3	26.0		26.3	32.9		25.5	28.6	
≥4	26.7	31.5		21.9	26.1		21.5	23.9	
Perceived physical health									
Fair to poor	31.8	33.2	.71	27.1	25.8	.73	16.9	14.1	.39
Good to excellent	68.2	66.8		72.9	74.2		83.1	85.9	
Perceived mental health									
Fair to poor	20.5	29.0	.01	13.8	16.5	.26	8.9	9.9	.62
Good to excellent	79.5	71.0		86.2	83.5		91.1	90.2	
Family having problems paying medical bills									
No	85.2	87.0	.63	86.8	84.1	.42	85.6	92.4	.009
Yes	14.8	13.0		13.2	15.9		14.4	7.6	
Have a usual source-of-care practitioner									
No	31.0	29.9	.82	29.1	29.7	.86	26.8	20.9	.06
Yes	69.0	70.1		70.9	70.3		73.2	79.1	
Census region									
Northeast	18.8	9.3	.12	16.2	15.6	.96	14.3	20.1	<.001
Midwest	20.1	29.6		16.2	17.7		4.8	19.2	
South	59.4	60.5		54.3	54.4		70.7	53.3	
West	1.7	0.5		13.3	12.3		10.2	7.4	
Census tract with high school education, mean (SD), %	35.4 (7.5)	35.1 (6.3)	.72	30.2 (8.7)	30.8 (9.7)	.56	20.7 (8.5)	22.2 (9.8)	.14
Census tract aged ≥65 y, mean (SD), %	12.5 (5.0)	12.7 (6.0)	.89	12.0 (5.3)	12.9 (7.0)	.16	12.6 (5.6)	13.9 (6.0)	.05

^a^ Data are presented as weighted percentages unless otherwise indicated.

^b^ *P* values were based on the Pearson χ^2^ statistic for categorical variables and the Wald test after fitting weighted linear regression for continuous variables.

^c^ Comorbidities include ever diagnoses of high blood pressure, heart disease, stroke, emphysema, chronic bronchitis (during the past 12 months), high cholesterol, cancer, diabetes, joint pain (during the past 12 months), arthritis, or asthma (having an episode during the past 12 months).

In the part 1 fully adjusted models of expenditure within each of the income-race ICE quintiles (Table 4; full models in eTable in Supplement 1), the odds of having any total health care expenditures were lower for Black respondents in Q3 (OR, 0.47; 95% CI, 0.30-0.73), Q4 (OR, 0.49; 95% CI, 0.28-0.87), and Q5 (OR, 0.44; 95% CI, 0.27-0.71), but these differences were driven by different expenditure categories (including less office-based, dental, and prescription drug spending) despite equal levels of health and comorbidities. The part 2 models suggest that among those who have any health care expenditures, Black respondents in Q2 spent significantly more than White respondents on outpatient expenditures (CR, 1.64; 95% CI, 1.08-2.47) but less on inpatient expenditures (CR, 0.62; 95% CI, 0.40-0.97). Black respondents spent less on office-based expenditures in Q4 (CR, 0.63; 95% CI, 0.46-0.85) and 30% less in total expenditures in Q5 (CR, 0.70; 95% CI, 0.56-0.86), driven by lower office-based expenditures (CR, 0.75; 95% CI, 0.58-0.98).

**Table 4.** Adjusted Odds Ratios of Having Any Expenditure and Cost Ratios of Positive Expenditure Comparing Black and White Medical Expenditure Panel Study Participants Living in the Same Index of Concentration at the Extremes (ICE) Income-Race Quintile (Q) From the 2-Part Model^a^

Variable	Expenditures						
	Total health care	Office based	Outpatient	Emergency department visit	Inpatient	Prescription drug	Dental care
**ICE income-race Q1 (low income, mostly Black people)**							
Part 1: odds ratio (95% CI) of having any expenditure	0.71 (0.41-1.21)	0.58 (0.39-0.88)	0.64 (0.39-1.05)	1.42 (0.89-2.26)	0.68 (0.41-1.13)	0.72 (0.50-1.03)	0.49 (0.34-0.70)
Part 2: cost ratio (95% CI) among persons with positive expenditure	0.91 (0.69-1.20)	0.82 (0.59-1.15)	1.32 (0.73-2.36)	0.87 (0.57-1.32)	1.24 (0.84-1.84)	0.66 (0.43-1.00)	1.32 (0.93-1.89)
**ICE income-race Q2**							
Part 1: odds ratio (95% CI) of having any expenditure	0.74 (0.46-1.21)	0.66 (0.44-0.97)	0.72 (0.47-1.12)	0.81 (0.55-1.18)	1.10 (0.64-1.86)	0.72 (0.48-1.07)	0.36 (0.26-0.51)
Part 2: cost ratio (95% CI) among persons with positive expenditure	0.83 (0.66-1.04)	0.77 (0.58-1.03)	1.64 (1.08-2.47)	1.20 (0.78-1.86)	0.62 (0.40-0.97)	0.98 (0.73-1.30)	1.01 (0.74-1.37)
**ICE income-race Q3**							
Part 1: odds ratio (95% CI) of having any expenditure	0.47 (0.30-0.73)	0.78 (0.57-1.06)	0.69 (0.42-1.12)	1.01 (0.69-1.48)	0.82 (0.49-1.38)	0.79 (0.56-1.11)	0.63 (0.44-0.89)
Part 2: cost ratio (95% CI) among persons with positive expenditure	1.06 (0.85-1.31)	0.96 (0.76-1.22)	1.12 (0.73-1.71)	0.88 (0.65-1.20)	1.36 (0.96-1.93)	1.07 (0.77-1.48)	1.14 (0.80-1.62)
**ICE income-race Q4**							
Part 1: odds ratio (95% CI) of having any expenditure	0.49 (0.28-0.87)	0.68 (0.45-1.03)	0.79 (0.51-1.23)	0.65 (0.43-0.98)	0.85 (0.49-1.47)	0.71 (0.49-1.03)	0.79 (0.54-1.16)
Part 2: cost ratio (95% CI) among persons with positive expenditure	0.86 (0.70-1.06)	0.63 (0.46-0.85)	1.10 (0.63-1.94)	0.96 (0.61-1.49)	1.01 (0.68-1.52)	0.80 (0.54-1.18)	1.06 (0.70-1.62)
**ICE income-race Q5 (high income, mostly White people)**							
Part 1: odds ratio (95% CI) of having any expenditure	0.44 (0.27-0.71)	0.57 (0.37-0.89)	0.98 (0.65-1.47)	1.52 (1.01-2.29)	0.97 (0.54-1.74)	0.58 (0.38-0.89)	0.53 (0.36-0.80)
Part 2: cost ratio (95% CI) among persons with positive expenditure	0.70 (0.56-0.86)	0.75 (0.58-0.98)	0.99 (0.55-1.79)	0.95 (0.62-1.43)	1.06 (0.64-1.78)	0.77 (0.57-1.05)	0.98 (0.70-1.37)

^a^ Results were adjusted for individual-level age, sex, educational level, family income level, employment, having any insurance, number of comorbidities, perceived physical health, perceived mental health, having a usual source-of-care practitioner, family having problems paying medical bills in the past 12 months, census tract–level percentage of population with high school education, and census tract–level percentage of population 65 years or older.

The Figure further quantifies the magnitude of the difference in annual spending in US dollar amounts in 2016. In total, Black respondents in the areas of highest White racial and economic privilege (Q5) spent $2145 less on total health care compared with White respondents in those same areas. The smallest differences in total expenditures ($79 annually; 95% CI, −$1187 to $1345) for Black and White respondents was in Q3, the most racially and economically integrated area.

**Figure.**
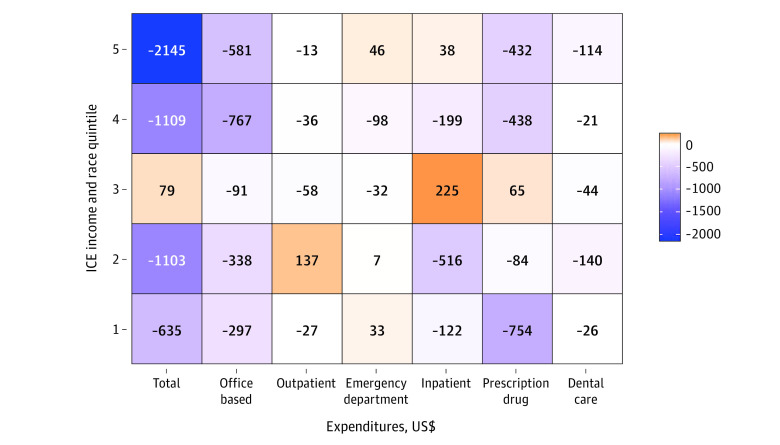
Adjusted Differences in Total and Type-Specific Health Care Expenditures Comparing Black and White Medical Expenditure Panel Survey Participants Living Within the Same Index of Concentration at the Extremes (ICE) Quintile in 2016 Results were adjusted for age, sex, educational level, family income level, employment, having any insurance, having a usual source-of-care practitioner, perceived physical health, perceived mental health, number of comorbidities, family having problems paying medical bills in the past 12 months, census tract–level percentage of population with high school education, and census tract–level percentage of population 65 years or older. The incremental expenditures comparing Black participants with White participants (ie, the marginal effects) are presented based on the combined part 1 and part 2 of the models. Quintile 5 reflects tracts that were mostly White and high income, whereas quintile 1 reflects tracts that were mostly Black and low income.

## Discussion

Our analysis sought to answer the question of whether differences in health care spending among Black and White adults would be minimized when Black and White adults lived under similar conditions. Our results offer 2 key takeaways: (1) differences in the uptake and amount of annual health care spending by Black and White adults were minimal in areas where Black and White adults lived under similar conditions of minimal racial and economic privilege; and (2) in contrast, Black adults had 56% lower odds of having any total health care expenditures in areas of mostly White high-income adults (driven largely by less office-based, dental, and prescription drug spending) and, among those with any expenditures, spent 30% less on health care. Black adults in areas of mostly White high-income adults had increased odds of having any ED expenditures and reduced odds of having prescription drug or dental care expenditures, likely driven by the significantly higher rates of uninsurance for Black adults in these areas. There was no significant difference in the amounts spent when looking only among Black and White people who had any expenditure—a proxy for equitable health care access. Altogether, our results suggest that expenditure disparities may disappear, but only under conditions of both racial and economic equity and equitable health care access.

Black adults who spent at all on health care spent equal to or significantly less than their White counterparts who lived under similar social and economic contexts, with the exception of higher outpatient expenditures in Q2. In the areas that were mostly high income with mostly White residents, this amounted to $2145 less spent annually by Black adults. It is possible that Black adults have reduced odds of health care expenditures because they are healthier and do not need the care. This hypothesis might be supported by the body of research suggesting that, due to long-standing disinvestment in Black neighborhoods, White people living in predominantly Black areas have poorer health than White people living in non-Black segregated areas.^14,15^ However, in the following paragraph, we point to several results to support that lower odds and amounts of health care spending are more likely because Black adults are missing out on care that they need.

In areas of extreme racial and economic deprivation or privilege (Q1 and Q5), Black and White adults had equally good physical health and similar comorbidities, yet Black adults still had reduced odds of any health expenditures and lower spending. In the most integrated areas (least deprivation or privilege extreme), Black adults had fewer comorbidities yet similar overall health spending. Among those who did any spending, Black adults in somewhat integrated areas (Q2) spent 64% more on outpatient care in the most integrated areas, but 38% less on inpatient expenditures, which might be explained by fewer comorbidities requiring extensive care. However, in the most integrated area (Q3), although findings of reduced odds of health care expenditures for Black compared with White respondents could be driven by these fewer comorbidities, the specific category of care driving reduced odds of having any health care expenditure was dental care, not inpatient or outpatient care as would be expected for comorbidities. Routine dental care is considered elective care and incurs high expenditures that those with few resources or reserves might forgo; low use of dental services may be a better marker of a health disparity than having healthy oral care.^16^ These findings work against the idea that reduced odds of spending by Black adults is due to better health, because even at equal levels of health, Black adults have lower odds of having health care expenditures than White adults. Rather, it may be that Black adults are forgoing care and may be underserved, despite being at equal health as White adults.

Lower odds of health care spending may be attributable to lower access to health insurance or poorer quality of insurance, which would be supported by our findings that most differences in health care expenditures disappeared when only looking at patients with any health care spending—a proxy for people who have entry to at least a minimum of health care. Furthermore, Black adults in the areas of highest White racial and economic privilege were 3 times as likely to be uninsured and have significantly lower income in those areas. Such low rates of insurance and lower economic resources likely mean lower use of health care, even when needed, because of affordability barriers. Several studies have suggested that insurance access remains a barrier to timely and affordable care, even after implementation of the Patient Protection and Affordable Care Act, and leads to avoiding or paying higher out-of-pocket costs for care.^17^

Even with insurance, Black adults often do not get high-value care and have fewer insurance options from which to choose,^18^ which may reflect systems of structural racism in health care. Because our study could not assess the reasons for health expenditures, we cannot know whether, for example, patients receiving low-value care at the ED could have otherwise received high-value primary care or whether their lack of primary care led to a more advanced condition that warranted ED care. A recent analysis found that Black Medicare beneficiaries are more likely than White beneficiaries to be admitted to a hospital or to seek care in an ED for conditions that would otherwise be managed through good primary care.^17^ The referral to low-value care is often a response to poor insurance coverage, which our findings also support in the thrice-higher rates of uninsured Black adults in Q5 (highest White racial and economic privilege), coinciding with lower use of office-based services and greater use of ED expenses. Another analysis found that areas with high Black concentrations had fewer insurers participating, possibly suggesting that even when Black residents of these areas have access to health insurance, there are fewer offerings of insurers and fewer physicians offering in-network services.^18^ Thus, similar to what we found for the area with the highest concentration of Black adults, higher expenditures on care may be due to less spending for office-based care with greater spending on ED care to compensate.

Another possible explanation for the lower expenditures for Black adults is that White adults are overusing care or receiving care at facilities that have higher costs for services. Although our study cannot assess the extent to which White patients are overcharged, previous work supports that White adults have more extensive use of health care services than Black adults,^19^ have greater health care spending even when under the same insurance plans and at equal levels of health as Black adults, and spend a greater proportion on primary care or specialty care than ED care.^20^ Although we cannot rule out the possibility of overuse or being charged at higher costs, the differences in distributions of socioeconomic position and health insurance for Black and White adults suggest that Black and White people in areas that are equal in racial and economic segregation may still live very differently and have different access to health care quality.

### Limitations

This study has some limitations. Although our study aimed to explore differences in health care expenditures in areas where Black and White people lived under similar conditions and expanded on previous studies by looking at both racial and economic segregation, our analysis may not fully capture how Black and White adults live in similar conditions. Our analysis was restricted to census tracts with 5% or more Black residents. In doing so, many White respondents were excluded, whereas Black respondents were more evenly distributed across the income-race quintiles; however, this criterion also yielded more Black respondents in the highest quintile, which we otherwise would not have been able to assess because of few Black respondents.

## Conclusions

Expanding on previous findings that health disparities between Black and White individuals are minimized or nearly nonexistent in areas where Black and White residents live under similar conditions, this cross-sectional study shows that health care expenditure disparities can also be minimized or nonexistent—but only under conditions of both racial and economic equity and equitable health care access. Altogether, our findings further reinforce that place is important and that the social, economic, and health care equity context is key to minimizing health care expenditure disparities for Black adults in the US.

## Notes

Supplement 1. **eFigure.** Flowchart of Study Sample **eTable.** Full Models: Adjusted Odds Ratio of Having Any Expenditure and Cost Ratio of Positive Expenditure Comparing Black MEPS Participants to White MEPS Participants Living in the Same ICE Income-Race Quintile From the Two-Part Model

Supplement 2.
Data Sharing Statement
