# Supplementary material for: Health Care Expenditures for Black and White US Adults Living Under Similar Conditions
Source: JAMA Health Forum. 2023 Nov 3;4(11):e233798. doi: 10.1001/jamahealthforum.2023.3798 (PMC10625039; doi:10.1001/jamahealthforum.2023.3798)
Supplement: Supplement 1. — eFigure. Flowchart of Study Sample eTable. Full Models: Adjusted Odds Ratio of Having Any Expenditure and Cost Ratio of Positive Expenditure Comparing Black MEPS Participants to White MEPS Participants Living in the Same ICE Income-Race Quintile From the Two-Part Model [file jamahealthforum-e233798-s001.pdf]

Supplementary Online Content

Dean LT, Zhang Y, McCleary RR, Dawit R, Thorpe RJ Jr, Gaskin D. Health care expenditures for Black and White US adults living under similar conditions. *JAMA Health Forum.* Published online November 3, 2023. doi:10.1001/jamahealthforum.2023.3798

eFigure. Flowchart of Study Sample

eTable. Full Models: Adjusted Odds Ratio of Having Any Expenditure and Cost Ratio of Positive Expenditure Comparing Black MEPS Participants to White MEPS Participants Living in the Same ICE Income-Race Quintile From the Two-Part Model

This supplementary material has been provided by the authors to give readers additional information about their work.

© 2023 Dean LT et al. *JAMA Health Forum.*

eFigure. Flowchart of Study Sample

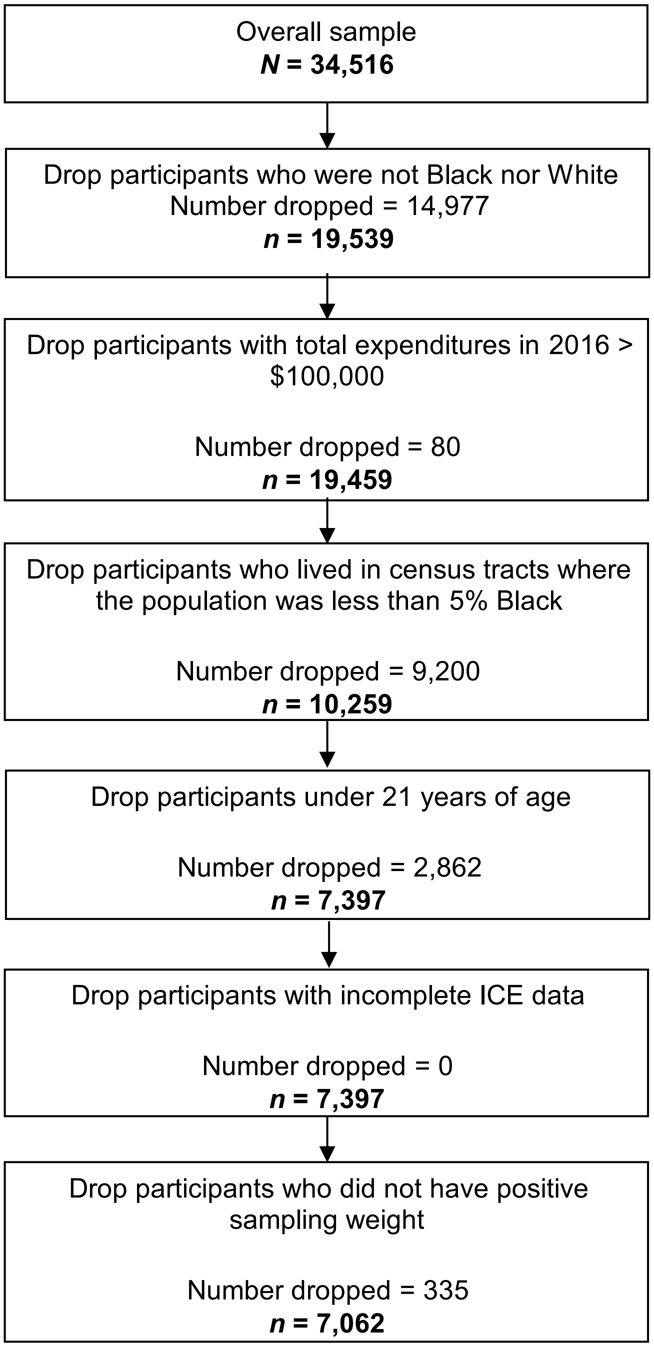

© 2023 Dean LT et al. *JAMA Health Forum.*

eTable. Full Models: Adjusted Odds Ratio of Having Any Expenditure and Cost Ratio of Positive Expenditure Comparing Black MEPS Participants to White MEPS Participants Living in the Same ICE Income-Race Quintile From the Two-Part Model

	**Total health care expenditures**	**Office-based expenditures**	**Outpatient expenditures**	**Emergency room visit expenditures**	**Inpatient expenditures**	**Prescription drug expenditures**	**Dental care expenditures**
**ICE Income-Race Q1 (low income, most Black people)**							
**Part I: odds ratio of having any expenditure**							
Race (NH Black)	0.71 (0.41, 1.21)	0.58 (0.38, 0.88)	0.64 (0.38, 1.05)	1.42 (0.89, 2.26)	0.68 (0.4, 1.13)	0.72 (0.50, 1.03)	0.49 (0.34, 0.70)
Age	0.99 (0.98, 1.01)	1.01 (1, 1.02)	1.01 (0.99, 1.02)	0.97 (0.96, 0.98)	1.00 (0.98, 1.01)	1.01 (1.00, 1.02)	0.99 (0.97, 1.00)
Female	2.09 (1.58, 2.77)	2.01 (1.52, 2.67)	1.13 (0.79, 1.61)	1.62 (1.2, 2.19)	1.42 (0.98, 2.05)	2.03 (1.55, 2.65)	1.56 (1.16, 2.11)
Years of education (12)	1.07 (0.72, 1.58)	1.64 (1.09, 2.45)	1.37 (0.91, 2.06)	0.96 (0.62, 1.51)	1.35 (0.76, 2.38)	1.16 (0.76, 1.78)	1.5 (0.98, 2.27)
Years of education (>12)	1.86 (1.18, 2.94)	2.44 (1.59, 3.74)	1.82 (1.13, 2.94)	1.16 (0.75, 1.78)	1.38 (0.77, 2.48)	1.64 (0.98, 2.75)	2.81 (1.75, 4.51)
Family income level (low/middle)	0.78 (0.55, 1.10)	0.69 (0.49, 0.97)	0.89 (0.59, 1.33)	0.75 (0.56, 1.01)	0.59 (0.39, 0.90)	0.60 (0.39, 0.90)	1.10 (0.78, 1.55)
Family income (high)	0.91 (0.51, 1.61)	1.07 (0.60, 1.92)	1.03 (0.55, 1.91)	0.65 (0.36, 1.19)	0.28 (0.11, 0.70)	0.61 (0.35, 1.06)	1.71 (1.17, 2.50)
Employment status (yes)	0.74 (0.48, 1.15)	1.06 (0.74, 1.53)	0.70 (0.45, 1.07)	0.81 (0.59, 1.09)	0.62 (0.42, 0.92)	1.00 (0.70, 1.41)	1.01 (0.69, 1.49)
Insurance status (insured)	2.48 (1.72, 3.57)	3.90 (2.51, 6.07)	3.97 (1.49, 10.61)	2.24 (1.33, 3.78)	4.46 (1.71, 11.57)	1.84 (1.15, 2.93)	1.65 (0.92, 2.93)
Have a usual source of care provider (yes)	3.64 (2.56, 5.18)	3.92 (2.87, 5.35)	1.34 (0.80, 2.23)	1.18 (0.79, 1.77)	1.34 (0.77, 2.31)	3.03 (2.15, 4.28)	2.67 (1.66, 4.27)
Family having problem paying medical bills in the past 12 months (yes)	1.47 (0.74, 2.91)	0.97 (0.61, 1.54)	1.18 (0.77, 1.80)	1.54 (1.01, 2.35)	0.88 (0.54, 1.44)	1.46 (0.85, 2.50)	1.28 (0.88, 1.87)
Perceived physical health (Good-excellent)	0.74 (0.47, 1.17)	0.63 (0.40, 0.99)	0.86 (0.53, 1.39)	0.57 (0.41, 0.80)	0.54 (0.36, 0.81)	0.70 (0.47, 1.04)	1.37 (0.91, 2.06)
Perceived mental Health (Good-excellent)	0.6 (0.35, 1.02)	0.67 (0.44, 1.03)	0.90 (0.58, 1.42)	0.70 (0.48, 1.03)	0.68 (0.43, 1.07)	0.59 (0.34, 1.02)	1.06 (0.66, 1.70)
Number of comorbidities (1)	2.72 (1.82, 4.06)	2.37 (1.54, 3.64)	1.05 (0.52, 2.13)	2.73 (1.48, 5.01)	0.90 (0.40, 2.04)	1.93 (1.25, 2.98)	1.71 (1.14, 2.58)

© 2023 Dean LT et al. *JAMA Health Forum.*

	Total health care expenditures	Office-based expenditures	Outpatient expenditures	Emergency room visit expenditures	Inpatient expenditures	Prescription drug expenditures	Dental care expenditures
Number of comorbidities (2-3)	4.11 (2.52, 6.71)	2.77 (1.87, 4.12)	1.62 (0.89, 2.95)	2.87 (1.66, 4.96)	1.33 (0.59, 3.00)	3.15 (2.15, 4.61)	1.20 (0.71, 2.02)
Number of comorbidities (>=4)	13.03 (6.84, 24.79)	6.61 (4.07, 10.74)	3.36 (1.75, 6.47)	4.44 (2.40, 8.24)	1.92 (0.91, 4.07)	13.84 (7.27, 26.34)	1.48 (0.84, 2.62)
Census-tract level % with high school education	2.49 (0.22, 27.4)	0.82 (0.1, 6.69)	0.04 (0.00, 0.70)	1.61 (0.19, 13.13)	1.76 (0.13, 22.55)	9.56 (1.30, 70.3)	2.86 (0.29, 27.83)
Census-tract level % with 65+ years of age	0.31 (0.01, 8.25)	4.61 (0.35, 60.16)	1.36 (0.03, 52.54)	0.22 (0.00, 5.25)	0.10 (0.00, 3.98)	0.81 (0.03, 18.90)	0.61 (0.03, 10.17)
**Part II: cost ratios among persons with positive expenditure**							
Race (NH Black)	0.91 (0.68, 1.20)	0.82 (0.59, 1.15)	1.32 (0.73, 2.36)	0.87 (0.57, 1.32)	1.24 (0.84, 1.84)	0.66 (0.43, 1.00)	1.32 (0.93, 1.89)
Age	1.00 (0.99, 1.01)	1.00 (0.99, 1.01)	1.01 (0.99, 1.03)	1.01 (0.99, 1.02)	0.99 (0.98, 1.00)	1.01 (1.00, 1.02)	1.00 (0.99, 1.01)
Female	0.98 (0.76, 1.25)	1.27 (0.96, 1.69)	0.88 (0.54, 1.42)	1.27 (0.89, 1.79)	0.82 (0.58, 1.16)	0.90 (0.71, 1.15)	1.16 (0.84, 1.59)
Years of education (12)	1.57 (1.23, 2.01)	1.37 (0.93, 2.03)	1.06 (0.44, 2.53)	1.32 (0.87, 2.00)	0.71 (0.44, 1.15)	2.52 (1.88, 3.39)	1.16 (0.73, 1.84)
Years of education (>12)	1.71 (1.24, 2.35)	1.73 (1.18, 2.53)	0.85 (0.36, 2.02)	1.36 (0.88, 2.08)	0.9.00 (0.52, 1.55)	1.79 (1.32, 2.43)	1.09 (0.69, 1.72)
Family income level (low/middle)	0.94 (0.74, 1.18)	1.19 (0.84, 1.69)	1.48 (0.71, 3.09)	1.31 (0.94, 1.84)	1.09 (0.76, 1.55)	1.11 (0.83, 1.48)	1.25 (0.85, 1.82)
Family income (high)	0.85 (0.64, 1.14)	1.73 (1.23, 2.43)	0.61 (0.26, 1.41)	1.38 (0.75, 2.51)	2.01 (0.96, 4.14)	0.99 (0.64, 1.51)	1.44 (0.71, 2.91)
Employment status (yes)	0.73 (0.56, 0.96)	0.66 (0.5, 0.88)	1.88 (0.97, 3.63)	1.91 (1.15, 3.14)	0.94 (0.61, 1.43)	0.51 (0.37, 0.69)	1.23 (0.89, 1.69)
Insurance status (insured)	2.57 (1.61, 4.13)	2.13 (1.19, 3.81)	1.15 (0.31, 4.33)	0.82 (0.42, 1.59)	5.57 (2.71, 11.41)	1.38 (0.64, 2.97)	2.15 (1.17, 3.94)
Have a usual source of care provider (yes)	1.38 (1.03, 1.84)	1.08 (0.77, 1.52)	1.26 (0.59, 2.71)	0.61 (0.42, 0.89)	1.08 (0.66, 1.74)	1.74 (1.16, 2.61)	0.77 (0.50, 1.21)
Family having problem paying medical bills in the past 12 months (yes)	1.10 (0.82, 1.46)	1.57 (1.02, 2.43)	0.96 (0.53, 1.74)	1.01 (0.71, 1.44)	0.73 (0.46, 1.16)	1.13 (0.79, 1.60)	1.08 (0.74, 1.58)
Perceived physical health (Good-excellent)	0.67 (0.51, 0.88)	1.01 (0.74, 1.38)	1.7 (0.86, 3.37)	0.83 (0.61, 1.14)	0.71 (0.48, 1.05)	0.64 (0.46, 0.88)	1.01 (0.75, 1.36)

© 2023 Dean LT et al. *JAMA Health Forum.*

	Total health care expenditures	Office-based expenditures	Outpatient expenditures	Emergency room visit expenditures	Inpatient expenditures	Prescription drug expenditures	Dental care expenditures
Perceived mental Health (Good-excellent)	0.71 (0.59, 0.86)	0.6 (0.44, 0.82)	0.38 (0.17, 0.84)	1.23 (0.86, 1.77)	0.84 (0.57, 1.22)	0.78 (0.55, 1.09)	1.04 (0.73, 1.50)
Number of comorbidities (1)	1.44 (0.92, 2.23)	0.96 (0.62, 1.48)	2.56 (0.77, 8.53)	1.51 (0.90, 2.53)	1.41 (0.61, 3.23)	1.14 (0.55, 2.34)	1.21 (0.72, 2.02)
Number of comorbidities (2-3)	1.84 (1.21, 2.78)	1.63 (1.08, 2.46)	4.70 (1.58, 13.93)	1.92 (1.15, 3.20)	1.19 (0.56, 2.49)	1.42 (0.83, 2.44)	0.71 (0.41), 1.25
Number of comorbidities (>=4)	2.57 (1.79, 3.67)	1.91 (1.22, 3.00)	2.45 (0.78, 7.69)	1.69 (0.93, 3.05)	1.25 (0.56, 2.78)	2.25 (1.3, 3.89)	1.24 (0.66, 2.35)
Census-tract level % with high school education	0.43 (0.07, 2.39)	0.68 (0.11, 4.53)	0.41 (0.01, 15.44)	0.58 (0.05, 6.10)	0.35 (0.05, 2.34)	3.52 (0.42, 29.49)	2.31 (0.32, 16.52)
Census-tract level % with 65+ years of age	0.11 (0.00, 1.73)	0.05 (0.00, 0.85)	0.02 (0.00, 8.42)	0.35 (0.02, 5.04)	0.01 (0.00, 0.27)	0.03 (0.00, 0.5)	2.45 (0.04, 129.54)
**ICE Income-Race Q2**							
**Part I: odds ratio of having any expenditure**							
Race (NH Black)	0.74 (0.46, 1.21)	0.66 (0.44, 0.97)	0.72 (0.47, 1.12)	0.81 (0.55, 1.18)	1.10 (0.64, 1.86)	0.72 (0.48, 1.07)	0.36 (0.26, 0.51)
Age	1.01 (0.99, 1.03	1.02 (1, 1.03)	1.01 (0.99, 1.02)	0.99 (0.97, 1.00)	1.01 (0.97, 1.02)	1.01 (0.99, 1.02)	0.99 (0.98, 1.01)
Female	2.48 (1.47, 4.18)	2.55 (1.73, 3.75)	1.37 (0.93, 2.02)	1.29 (0.91, 1.83)	1.80 (1.09, 2.98)	1.49 (0.91, 2.42)	1.33 (0.92, 1.91)
Years of education (12)	0.51 (0.21, 1.23)	0.80 (0.42, 1.54)	0.83 (0.46, 1.48)	0.92 (0.54, 1.54)	0.6 (0.32, 1.12)	0.68 (0.36, 1.31)	0.68 (0.38, 1.21)
Years of education (>12)	0.96 (0.39, 2.33)	1.85 (1.01, 3.38)	0.96 (0.51, 1.83)	0.85 (0.49, 1.45)	0.57 (0.31, 1.04)	1.5 (0.79, 2.86)	1.23 (0.72, 2.12)
Family income level (low/middle)	1.09 (0.58, 2.04)	1.07 (0.63, 1.81)	0.74 (0.43, 1.26)	1.18 (0.73, 1.89)	0.67 (0.34, 1.33)	1.13 (0.65, 1.95)	1.12 (0.73, 1.71)
Family income (high)	1.48 (0.65, 3.39)	1.56 (0.81, 3.03)	1.38 (0.80, 2.38)	1.06 (0.60, 1.86)	0.78 (0.33, 1.85)	1.16 (0.58, 2.32)	1.71 (0.96, 3.02)
Employment status (yes)	1.73 (0.91, 3.28)	0.92 (0.53, 1.58)	0.71 (0.39, 1.22)	0.87 (0.55, 1.38)	0.69 (0.38, 1.28)	0.78 (0.48, 1.28)	1.33 (0.87, 2.03)
Insurance status (insured)	4.89 (2.05, 11.63)	3.92 (1.95, 7.89)	3.38 (0.97, 11.7)	1.54 (0.56, 4.25)	0.47 (0.23, 0.97)	3.25 (1.66, 6.35)	3.36 (1.44, 7.81)
Have a usual source of care provider (yes)	2.85 (1.69, 4.80)	2.79 (1.83, 4.26)	3.69 (1.98, 6.87)	1.02 (0.56, 1.85)	1.75 (0.85, 3.61)	2.84 (1.74, 4.62)	1.92 (1.10, 3.36)
Family having problem paying medical bills in	4.06 (1.84, 8.92)	2.19 (1.19, 4.00)	1.31 (0.79, 2.18)	1.69 (1.01, 2.83)	1.53 (0.8, 2.91)	2.86 (1.57, 5.22)	0.87 (0.51, 1.51)

© 2023 Dean LT et al. *JAMA Health Forum.*

	Total health care expenditures	Office-based expenditures	Outpatient expenditures	Emergency room visit expenditures	Inpatient expenditures	Prescription drug expenditures	Dental care expenditures
the past 12 months (yes)							
Perceived physical health (Good-excellent)	0.42 (0.18, 0.95)	0.45 (0.26, 0.79)	0.92 (0.57, 1.49)	0.73 (0.43, 1.26)	0.49 (0.23, 1.07)	0.62 (0.33, 1.15)	1.75 (0.96, 3.18)
Perceived mental Health (Good-excellent)	0.45 (0.12, 1.66)	0.79 (0.37, 1.69)	0.74 (0.42, 1.31)	0.58 (0.32, 1.04)	0.85 (0.44, 1.64)	0.34 (0.13, 0.84)	0.95 (0.49, 1.83)
Number of comorbidities (1)	2.58 (1.41, 4.72)	2.36 (1.28, 4.32)	1.98 (0.67, 5.81)	1.9 (0.77, 4.66)	0.74 (0.27, 1.97)	2.8 (1.75, 4.47)	1.03 (0.56, 1.91)
Number of comorbidities (2-3)	5.82 (2.9, 11.69)	3.47 (1.7, 7.09)	2.34 (0.92, 5.97)	2.14 (1.1, 4.14)	0.91 (0.32, 2.56)	8.39 (4.35, 16.17)	1.94 (0.99, 3.81)
Number of comorbidities (>=4)	23.52 (6.28, 88.00)	10.36 (4.47, 24.01)	2.77 (0.91, 8.49)	4.12 (2.19, 7.78)	1.86 (0.69, 5.02)	19.9 (8.85, 44.73)	1.95 (0.94, 4.01)
Census-tract level % with high school education	0.08 (0.01, 2.03)	0.33 (0.04, 2.88)	4.51 (0.15, 132.71)	0.51 (0.05, 4.56)	1.88 (0.07, 46.66)	5.83 (0.33, 100.42)	0.01 (0.00, 0.22)
Census-tract level % with 65+ years of age	89.7 (1.19, 6718.39)	4.55 (0.19, 104.92)	4.2 (0.05, 318.25)	0.44 (0.01, 12.79)	0.88 (0.01, 61.45)	2.62 (0.05, 128.04)	8.41 (0.5, 141.37)
**Part II: cost ratios among persons with positive expenditure**							
Race (NH Black)	0.83 (0.66, 1.04)	0.77 (0.58, 1.03)	1.64 (1.08, 2.47)	1.20 (0.78, 1.86)	0.62 (0.40, 0.97)	0.98 (0.73, 1.30)	1.01 (0.74, 1.37)
Age	1.00 (0.99, 1.01)	1.00 (0.99, 1.01)	1.00 (0.98, 1.02)	0.97 (0.95, 0.99)	1.00 (0.99, 1.02)	1.01 (1.00, 1.02)	0.99 (0.98, 1.01)
Female	1.16 (0.96, 1.41)	1.25 (0.98, 1.58)	0.79 (0.48, 1.32)	0.63 (0.45, 0.87)	0.81 (0.53, 1.23)	0.98 (0.72, 1.33)	1.01 (0.74, 1.39)
Years of education (12)	1.07 (0.79, 1.44)	1.27 (0.94, 1.72)	1.03 (0.37, 2.88)	0.96 (0.59, 1.55)	2.29 (1.10, 4.74)	0.85 (0.57, 1.27)	0.41 (0.25, 0.66)
Years of education (>12)	1.25 (0.91, 1.73)	1.14 (0.84, 1.55)	1.25 (0.46, 3.35)	0.85 (0.55, 1.30)	1.29 (0.61, 2.74)	1.55 (1.03, 2.34)	0.51 (0.29, 0.88)
Family income level (low/middle)	0.84 (0.59, 1.21)	1.42 (1.10, 1.84)	0.45 (0.24, 0.83)	1.71 (1.25, 2.34)	1.14 (0.64, 2.02)	0.77 (0.52, 1.15)	1.09 (0.74, 1.61)
Family income (high)	1.04 (0.71, 1.54)	1.59 (1.10, 2.29)	1.01 (0.45, 2.31)	1.68 (0.93, 3.04)	0.92 (0.55, 1.54)	0.81 (0.5, 1.29)	1.72 (1.08, 2.73)
Employment status (yes)	0.62 (0.47, 0.82)	0.74 (0.54, 1.01)	1.37 (0.74, 2.53)	1.06 (0.66, 1.71)	1.05 (0.55, 1.97)	0.67 (0.44, 1.00)	1.13 (0.75, 1.68)
Insurance status (insured)	0.96 (0.58, 1.59)	1.29 (0.73, 2.27)	3.43 (1.41, 8.36)	0.46 (0.15, 1.38)	2.3 (1.23, 4.31)	0.66 (0.34, 1.31)	1.38 (0.77, 2.47)

© 2023 Dean LT et al. *JAMA Health Forum.*

	Total health care expenditures	Office-based expenditures	Outpatient expenditures	Emergency room visit expenditures	Inpatient expenditures	Prescription drug expenditures	Dental care expenditures
Have a usual source of care provider (yes)	1.34 (0.99, 1.82)	1.24 (0.91, 1.69)	1.88 (0.61, 5.78)	1.75 (1.11, 2.75)	0.52 (0.23, 1.17)	1.05 (0.58, 1.88)	1.39 (0.91, 2.12)
Family having problem paying medical bills in the past 12 months (yes)	1.20 (0.91, 1.59)	1.25 (0.92, 1.71)	1.88 (0.84, 4.19)	1.09 (0.72, 1.65)	0.75 (0.52, 1.09)	0.82 (0.59, 1.15)	1.11 (0.68, 1.81)
Perceived physical health (Good-excellent)	0.44 (0.31, 0.62)	0.68 (0.46, 1.00)	0.31 (0.15, 0.63)	0.74 (0.48, 1.15)	0.36 (0.21, 0.61)	0.58 (0.41, 0.83)	1.12 (0.69, 1.81)
Perceived mental Health (Good-excellent)	0.84 (0.61, 1.18)	1.15 (0.79, 1.69)	1.55 (0.81, 2.95)	1.00 (0.71, 1.41)	2.18 (1.24, 3.84)	0.42 (0.28, 0.63)	0.83 (0.55, 1.24)
Number of comorbidities (1)	0.89 (0.61, 1.31)	0.97 (0.61, 1.52)	0.96 (0.24, 3.75)	1.39 (0.85, 2.26)	1.67 (0.60, 4.66)	0.54 (0.26, 1.11)	1.09 (0.55, 2.15)
Number of comorbidities (2-3)	1.32 (0.88, 1.96)	1.12 (0.73, 1.71)	1.46 (0.51, 4.26)	1.40 (0.82, 2.39)	0.98 (0.33, 2.87)	1.09 (0.58, 2.02)	1.21 (0.74, 1.96)
Number of comorbidities (>=4)	1.99 (1.29, 3.08)	1.80 (1.09, 2.95)	1.08 (0.39, 2.97)	1.46 (0.72, 2.95)	1.53 (0.48, 4.89)	2.07 (0.96, 4.45)	1.14 (0.60, 2.17)
Census-tract level % with high school education	0.31 (0.07, 1.39)	0.36 (0.07, 1.74)	0.16 (0.00, 4.92)	3.27 (0.37, 28.24)	0.04 (0.00, 0.57)	0.98 (0.11, 8.41)	1.48 (0.19, 11.26)
Census-tract level % with 65+ years of age	0.49 (0.03, 7.21)	2.51 (0.26, 23.76)	0.01 (0.00, 0.22)	0.17 (0.01, 2.34)	0.15 (0.01, 2.55)	0.17 (0.01, 2.27)	0.12 (0.02, 2.07)
**ICE Income-Race Q3**							
**Part I: odds ratio of having any expenditure**							
Race (NH Black)	0.47 (0.30, 0.73)	0.78 (0.57, 1.06)	0.69 (0.42, 1.12)	1.01 (0.69, 1.48)	0.82 (0.49, 1.38)	0.79 (0.56, 1.11)	0.63 (0.44, 0.89)
Age	1.01 (0.99, 1.03)	1.02 (1.01, 1.04)	1.02 (1.00, 1.03)	0.99 (0.97, 1.00)	1.01 (0.99, 1.02)	1.02 (1.01, 1.04)	0.99 (0.98, 1.01)
Female	2.53 (1.52, 4.21)	1.77 (1.21, 2.60)	1.7 (1.09, 2.63)	1.02 (0.67, 1.56)	1.33 (0.78, 2.25)	1.52 (1.02, 2.27)	2.19 (1.51, 3.22)
Years of education (12)	0.77 (0.35, 1.69)	1.03 (0.58, 1.80)	1.17 (0.64, 2.14)	0.83 (0.47, 1.47)	1.02 (0.48, 2.17)	0.78 (0.40, 1.50)	0.94 (0.47, 1.87)
Years of education (>12)	2.37 (0.99, 5.66)	2.07 (1.13, 3.79)	1.2 (0.64, 2.25)	0.88 (0.49, 1.57)	1.11 (0.51, 2.37)	1.45 (0.71, 2.98)	2.02 (0.97, 4.19)
Family income level (low/middle)	1.03 (0.46, 2.31)	1.38 (0.82, 2.31)	0.67 (0.36, 1.23)	0.71 (0.41, 1.20)	0.71 (0.39, 1.29)	0.92 (0.50, 1.69)	0.88 (0.55, 1.40)
Family income (high)	1.18 (0.55, 2.55)	1.08 (0.55, 2.15)	0.54 (0.25, 1.16)	0.63 (0.31, 1.29	0.64 (0.22, 1.81)	0.81 (0.41, 1.59)	1.52 (0.88, 2.63)

© 2023 Dean LT et al. *JAMA Health Forum.*

	Total health care expenditures	Office-based expenditures	Outpatient expenditures	Emergency room visit expenditures	Inpatient expenditures	Prescription drug expenditures	Dental care expenditures
Employment status (yes)	0.79 (0.37, 1.65)	1.01 (0.59, 1.69)	0.71 (0.4, 1.23)	0.98 (0.55, 1.76	0.73 (0.38, 1.39	0.84 (0.46, 1.53	1.14 (0.73, 1.78
Insurance status (insured)	2.22 (1.06, 4.62)	1.97 (0.87, 4.46)	1.61 (0.48, 5.37)	1.15 (0.48, 2.76)	1.18 (0.37, 3.72)	3.91 (1.64, 9.25)	2.13 (1.03, 4.41)
Have a usual source of care provider (yes)	2.51 (1.57, 3.96)	2.08 (1.45, 3.00)	2.14 (1.14, 4.02)	1.06 (0.66, 1.69)	0.67 (0.34, 1.34)	2.49 (1.69, 3.66)	1.31 (0.86, 2.01)
Family having problem paying medical bills in the past 12 months (yes)	1.01 (0.47, 2.14)	1.27 (0.7, 2.32)	1.75 (0.96, 3.19)	1.36 (0.79, 2.36)	1.72 (0.78, 3.78)	1.78 (0.84, 3.77)	1.04 (0.66, 1.64)
Perceived physical health (Good-excellent)	1.09 (0.52, 2.28)	0.73 (0.42, 1.25)	0.79 (0.44, 1.41)	0.38 (0.21, 0.70)	0.21 (0.12, 0.37)	0.68 (0.36, 1.27)	1.60 (1.06, 2.43)
Perceived mental Health (Good-excellent)	1.05 (0.36, 3.03)	0.71 (0.34, 1.48)	1.73 (0.98, 3.07)	0.60 (0.32, 1.10)	1.22 (0.57, 2.59)	0.64 (0.26, 1.60)	0.95 (0.50, 1.78)
Number of comorbidities (1)	2.09 (1.22, 3.57)	1.61 (0.97, 2.67)	0.67 (0.24, 1.86)	1.9 (0.89, 4.06)	0.95 (0.29, 3.08)	2.34 (1.32, 4.16)	0.95 (0.55, 1.63)
Number of comorbidities (2-3)	5.21 (2.63, 10.31)	2.81 (1.55, 5.12)	2.14 (0.97, 4.70)	3.13 (1.55, 6.32)	1.02 (0.38, 2.69)	4.28 (2.54, 7.19)	1.13 (0.68, 1.85)
Number of comorbidities (>=4)	16.4 (4.17, 64.51)	4.31 (1.99, 9.35)	2.24 (0.93, 5.38)	3.83 (1.86, 7.89)	1.55 (0.54, 4.44)	11.7 (5.14, 26.62)	1.48 (0.82, 2.68)
Census-tract level % with high school education	0.40 (0.02, 6.00)	1.91 (0.19, 19.27)	3.04 (0.15, 58.2)	0.43 (0.03, 5.73)	4.23 (0.14, 126.68)	0.27 (0.01, 4.47)	0.11 (0.01, 0.89
Census-tract level % with 65+ years of age	0.19 (0.01, 4.07)	1.69 (0.05, 48.70)	0.43 (0.01, 15.47)	5.81 (0.36, 92.14)	0.21 (0.01, 15.52)	34.89 (1.76, 690.26)	4.50 (0.36, 54.96)
**Part II: cost ratios among persons with positive expenditure**							
Race (NH Black)	1.06 (0.85, 1.31)	0.96 (0.76, 1.22)	1.12 (0.73, 1.71)	0.88 (0.65, 1.20)	1.36 (0.96, 1.93)	1.07 (0.77, 1.48)	1.14 (0.80, 1.62)
Age	1.00 (0.99, 1.01)	1.00 (0.99, 1.01)	0.97 (0.95, 0.98)	0.99 (0.98, 1.00)	0.99 (0.97, 1.00)	1.00 (0.99, 1.01)	1.02 (1, 1.03)
Female	1.19 (0.91, 1.55)	1.06 (0.86, 1.31)	0.70 (0.43, 1.13)	1.22 (0.92, 1.60)	0.91 (0.52, 1.62)	1.05 (0.74, 1.49)	1.06 (0.75, 1.49)
Years of education (12)	0.83 (0.54, 1.27)	1.58 (1.17, 2.13)	0.93 (0.45, 1.92)	1.29 (0.84, 1.97)	0.64 (0.37, 1.12)	0.92 (0.57, 1.49)	0.80 (0.41, 1.59)
Years of education (>12)	0.96 (0.64, 1.45)	1.99 (1.45, 2.72)	0.86 (0.49, 1.50)	1.51 (1.02, 2.22)	1.20 (0.67, 2.14)	1.05 (0.68, 1.63)	1.03 (0.55, 1.93)

© 2023 Dean LT et al. *JAMA Health Forum.*

	Total health care expenditures	Office-based expenditures	Outpatient expenditures	Emergency room visit expenditures	Inpatient expenditures	Prescription drug expenditures	Dental care expenditures
Family income level (low/middle)	0.86 (0.62, 1.21)	1.23 (0.88, 1.72)	1.89 (1.03, 3.46)	1.29 (0.88, 1.91)	1.00 (0.63, 1.57)	0.77 (0.55, 1.07)	1.21 (0.71, 2.09)
Family income (high)	1.06 (0.71, 1.59)	1.42 (0.98, 2.05)	2.75 (1.18, 6.39)	1.25 (0.77, 2.02)	1.04 (0.57, 1.88)	0.68 (0.45, 1.02)	1.08 (0.65, 1.77)
Employment status (yes)	0.69 (0.51, 0.95)	0.64 (0.46, 0.89)	0.48 (0.23, 0.99)	0.97 (0.60, 1.55)	1.14 (0.67, 1.94)	0.73 (0.51, 1.07)	0.74 (0.44, 1.24)
Insurance status (insured)	2.53 (1.54, 4.14)	1.71 (0.91, 3.21)	3.71 (1.95, 7.06)	2.15 (1.29, 3.59)	1.48 (0.31, 7.01)	2.02 (1.17, 3.50)	2.96 (2.00, 4.38)
Have a usual source of care provider (yes)	1.01 (0.66, 1.52)	1.51 (1.10, 2.08)	0.96 (0.49, 1.87)	0.97 (0.65, 1.43)	0.94 (0.55, 1.61)	2.29 (1.57, 3.36)	0.52 (0.32, 0.85)
Family having problem paying medical bills in the past 12 months (yes)	1.52 (1.13, 2.07)	1.47 (1.02, 2.13)	1.45 (0.83, 2.56)	1.25 (0.81, 1.93)	0.63 (0.38, 1.04)	1.36 (0.93, 1.98)	2.85 (1.58, 5.13)
Perceived physical health (Good-excellent)	0.38 (0.27, 0.54)	0.73 (0.56, 0.93)	0.99 (0.56, 1.73)	0.86 (0.61, 1.20)	0.67 (0.46, 0.96)	0.94 (0.71, 1.26)	0.51 (0.34, 0.77)
Perceived mental Health (Good-excellent)	1.25 (0.93, 1.68)	1.13 (0.84, 1.53)	1.61 (0.93, 2.76)	1.24 (0.93, 1.66)	0.99 (0.59, 1.66)	0.69 (0.51, 0.96)	2.05 (1.18, 3.54)
Number of comorbidities (1)	0.98 (0.58, 1.65)	1.46 (1.04, 2.05)	3.02 (1.15, 7.94)	0.72 (0.43, 1.21)	0.66 (0.24, 1.79)	1.35 (0.77, 2.38)	1.74 (0.93, 3.24)
Number of comorbidities (2-3)	1.57 (0.94, 2.61)	2.01 (1.44, 2.81)	2.96 (1.45, 6.07)	0.98 (0.61, 1.57)	0.61 (0.31, 1.21)	4.58 (2.66, 7.86)	1.07 (0.67, 1.70)
Number of comorbidities (>=4)	2.59 (1.56, 4.29)	2.87 (1.96, 4.21)	2.63 (1.25, 5.52)	0.89 (0.48, 1.63)	1.01 (0.46, 2.20)	5.91 (3.30, 10.59)	1.17 (0.59, 2.30)
Census-tract level % with high school education	5.21 (1.38, 19.56)	1.13 (0.31, 4.07)	0.63 (0.03, 12.72)	1.87 (0.29, 11.74)	2.57 (0.45, 14.6)	23.24 (4.27, 126.51)	0.38 (0.06, 2.33)
Census-tract level % with 65+ years of age	0.89 (0.21, 3.80)	2.55 (0.3, 21.67)	1.59 (0.02, 91.14)	2.46 (0.51, 11.83)	4.09 (0.55, 30.42)	0.29 (0.04, 1.89)	0.31 (0.05, 1.55)
**ICE Income-Race Q4**							
**Part I: odds ratio of having any expenditure**							
Race (NH Black)	0.49 (0.28, 0.87)	0.68 (0.45, 1.03)	0.79 (0.51, 1.23)	0.65 (0.43, 0.98)	0.85 (0.49, 1.47)	0.71 (0.49, 1.03)	0.79 (0.54, 1.16)
Age	1.00 (0.98, 1.02)	1.00 (0.98, 1.02)	1.00 (0.98, 1.02)	1.00 (0.98, 1.01)	0.99 (0.98, 1.01)	1.00 (0.98, 1.01)	1.00 (0.99, 1.01)

© 2023 Dean LT et al. *JAMA Health Forum.*

	Total health care expenditures	Office-based expenditures	Outpatient expenditures	Emergency room visit expenditures	Inpatient expenditures	Prescription drug expenditures	Dental care expenditures
Female	3.80 (2.43, 5.92)	3.55 (2.57, 4.91)	1.21 (0.86, 1.69)	1.77 (1.27, 2.49)	1.78 (1.2, 2.66)	2.38 (1.64, 3.44)	1.40 (1.14, 1.72)
Years of education (12)	2.65 (1.07, 6.55)	1.95 (0.88, 4.30)	1.40 (0.69, 2.84)	1.44 (0.70, 2.95)	1.23 (0.56, 2.66)	1.54 (0.71, 3.36)	1.96 (1.25, 3.07)
Years of education (>12)	2.25 (0.89, 5.66)	2.02 (0.92, 4.44)	1.36 (0.71, 2.61)	1.37 (0.67, 2.80)	1.27 (0.58, 2.77)	1.54 (0.73, 3.28)	2.71 (1.69, 4.32)
Family income level (low/middle)	0.71 (0.32, 1.55)	1.06 (0.61, 1.88)	1.27 (0.73, 2.21)	0.70 (0.41, 1.20)	1.22 (0.66, 2.25)	1.33 (0.72, 2.46)	0.88 (0.56, 1.37)
Family income (high)	1.11 (0.56, 2.17)	1.98 (1.15, 3.39)	1.56 (0.82, 2.98)	0.52 (0.29, 0.95)	0.86 (0.41, 1.81)	1.32 (0.78, 2.23)	1.29 (0.78, 2.14)
Employment status (yes)	0.67 (0.28, 1.57)	0.72 (0.40, 1.28)	0.75 (0.45, 1.25)	1.81 (1.09, 3.01)	0.91 (0.49, 1.63)	0.75 (0.42, 1.34)	0.93 (0.67, 1.29)
Insurance status (insured)	2.53 (1.20, 5.30)	3.33 (1.89, 5.87)	1.78 (0.69, 4.57)	1.38 (0.63, 3.00)	2.27 (0.70, 7.33)	1.95 (0.95, 4.01)	4.13 (1.77, 9.63)
Have a usual source of care provider (yes)	2.49 (1.47, 4.20)	2.41 (1.63, 3.56)	2.55 (1.52, 4.27)	0.99 (0.61, 1.59)	0.61 (0.31, 1.18)	2.76 (1.87, 4.05)	1.45 (1.01, 2.10)
Family having problem paying medical bills in the past 12 months (yes)	2.04 (0.95, 4.38)	1.71 (1.04, 2.80)	1.59 (0.92, 2.74)	1.49 (0.91, 2.41)	1.38 (0.69, 2.75)	2.12 (1.13, 4.27)	1.16 (0.74, 1.82)
Perceived physical health (Good-excellent)	0.90 (0.40, 2.02)	0.75 (0.41, 1.38)	0.65 (0.44, 0.96)	0.50 (0.31, 0.81)	0.87 (0.51, 1.51)	0.85 (0.51, 1.44)	1.44 (0.92, 2.26)
Perceived mental Health (Good-excellent)	0.56 (0.19, 1.61)	0.64 (0.37, 1.11)	0.96 (0.58, 1.57)	0.82 (0.48, 1.38)	0.52 (0.28, 0.94)	0.48 (0.23, 0.99)	1.37 (0.88, 2.13)
Number of comorbidities (1)	1.62 (0.83, 3.15)	1.64 (1.04, 2.59)	1.38 (0.66, 2.87)	1.04 (0.52, 2.08)	1.23 (0.59, 2.53)	1.92 (1.29, 2.84)	1.16 (0.79, 1.72)
Number of comorbidities (2-3)	6.42 (2.78, 14.81)	3.53 (1.9, 6.54)	2.52 (1.27, 4.98)	1.26 (0.66, 2.40)	1.26 (0.64, 2.48)	5.69 (3.19, 10.14)	1.62 (1.01, 2.59)
Number of comorbidities (>=4)	12.52 (2.57, 61.02)	6.14 (2.42, 15.55)	2.85 (1.37, 5.95)	2.12 (1.10, 4.11)	4.20 (1.97, 8.95)	29.05 (11.31, 74.59)	1.43 (0.85, 2.41)
Census-tract level % with high school education	0.06 (0.00, 1.43)	0.33 (0.02, 4.35)	2.98 (0.25, 35.46)	1.40 (0.16, 12.12)	0.71 (0.07, 7.03)	0.13 (0.01, 1.29)	0.31 (0.03, 3.21)
Census-tract level % with 65+ years of age	0.11 (0.01, 12.63)	0.12 (0.00, 4.25)	3.04 (0.12, 71.8)	7.34 (0.35, 150.62)	1.07 (0.01, 57.78)	4.39 (0.21, 88.17)	2.48 (0.12, 49.84)
**Part II: cost ratios among persons with positive expenditure**							

© 2023 Dean LT et al. *JAMA Health Forum.*

	Total health care expenditures	Office-based expenditures	Outpatient expenditures	Emergency room visit expenditures	Inpatient expenditures	Prescription drug expenditures	Dental care expenditures
Race (NH Black)	0.86 (0.70, 1.06)	0.63 (0.46, 0.85)	1.10 (0.63, 1.94)	0.96 (0.61, 1.49)	1.01 (0.68, 1.52)	0.80 (0.54, 1.18)	1.06 (0.70, 1.62)
Age	1.00 (0.99, 1.01)	1.00 (0.99, 1.01)	0.96 (0.95, 0.98)	1.00 (0.99, 1.02)	0.98 (0.97, 1.00)	1.01 (0.99, 1.02)	1.01 (1.00, 1.03)
Female	1.35 (1.11, 1.64)	1.28 (1.04, 1.57)	0.95 (0.62, 1.46)	1.02 (0.75, 1.38)	0.87 (0.65, 1.18)	1.18 (0.83, 1.67)	1.19 (0.81, 1.74)
Years of education (12)	1.10 (0.76, 1.59)	1.66 (1.14, 2.39)	0.65 (0.27, 1.51)	1.38 (0.89, 2.13)	0.85 (0.57, 1.27)	1.27 (0.88, 1.85)	0.91 (0.53, 1.5)2
Years of education (>12)	1.33 (0.94, 1.90)	2.19 (1.56, 3.07)	0.73 (0.33, 1.61)	1.53 (0.96, 2.43)	0.69 (0.47, 1.01)	1.81 (1.22, 2.70)	1.01 (0.62, 1.61)
Family income level (low/middle)	0.93 (0.65, 1.34)	0.93 (0.71, 1.23)	1.49 (0.84, 2.66)	0.81 (0.52, 1.23)	0.81 (0.43, 1.52)	0.63 (0.40, 0.99)	1.48 (0.95, 2.29)
Family income (high)	0.91 (0.63, 1.31)	0.96 (0.63, 1.46)	1.26 (0.60, 2.61)	0.84 (0.50, 1.42)	0.68 (0.33, 1.38)	0.95 (0.57, 1.57)	1.36 (0.86, 2.17)
Employment status (yes)	0.99 (0.71, 1.40)	0.91 (0.66, 1.26)	1.13 (0.63, 2.02)	1.61 (1.10, 2.36)	0.87 (0.57, 1.34)	0.96 (0.69, 1.35)	1.64 (1.18, 2.29)
Insurance status (insured)	1.43 (0.59, 3.44)	1.79 (0.86, 3.70)	14.39 (6.7, 30.88)	0.39 (0.21, 0.72)	1.01 (0.35, 2.89)	0.88 (0.27, 2.88)	1.74 (0.57, 5.26)
Have a usual source of care provider (yes)	1.21 (0.87, 1.68)	1.31 (0.96, 1.80)	2.21 (1.15, 4.24)	1.47 (0.99, 2.19)	1.28 (0.84, 1.94)	1.90 (1.16, 3.11)	0.89 (0.54, 1.48)
Family having problem paying medical bills in the past 12 months (yes)	1.12 (0.88, 1.43)	1.25 (0.94, 1.65)	1.70 (0.87, 3.34)	0.85 (0.58, 1.24)	0.82 (0.55, 1.22)	0.97 (0.70, 1.34)	1.15 (0.69, 1.92)
Perceived physical health (Good-excellent)	0.8 (0.62, 1.03)	0.74 (0.52, 1.06)	1.03 (0.56, 1.91)	0.83 (0.57, 1.21)	1.30 (0.93, 1.82)	0.56 (0.41, 0.76)	0.97 (0.62, 1.52)
Perceived mental Health (Good-excellent)	0.75 (0.58, 0.97)	1.03 (0.76, 1.41)	1.3 (0.69, 2.44)	1.25 (0.86, 1.82)	1.10 (0.72, 1.68)	0.64 (0.45, 0.92)	0.75 (0.45, 1.25)
Number of comorbidities (1)	1.65 (1.12, 2.44)	1.31 (0.86, 1.96)	0.88 (0.41, 1.88)	1.13 (0.58, 2.18)	2.14 (1.26, 3.61)	1.60 (0.98, 2.60)	1.05 (0.61, 1.82)
Number of comorbidities (2-3)	2.35 (1.68, 3.27)	1.94 (1.24, 3.05)	1.27 (0.69, 2.34)	1.3 (0.87, 1.93)	2.62 (1.55, 4.43)	2.90 (1.81, 4.67)	0.91 (0.56, 1.49)
Number of comorbidities (>=4)	4.59 (3.07, 6.87)	2.44 (1.50, 3.97)	3.06 (1.4, 6.71)	1.05 (0.57, 1.92)	3.16 (2.01, 5.00)	3.41 (2.10, 5.53)	1.31 (0.69, 2.47)
Census-tract level % with high school education	0.9 (0.24, 3.32)	1.75 (0.38, 7.90)	0.49 (0.07, 3.22)	0.93 (0.10, 8.38)	0.29 (0.04, 2.11)	0.76 (0.13, 4.33)	0.36 (0.06, 2.01)

© 2023 Dean LT et al. *JAMA Health Forum.*

	Total health care expenditures	Office-based expenditures	Outpatient expenditures	Emergency room visit expenditures	Inpatient expenditures	Prescription drug expenditures	Dental care expenditures
Census-tract level % with 65+ years of age	1.84 (0.27, 12.25)	1.91 (0.24, 15.18)	0.85 (0.05, 13.61)	0.39 (0.03, 4.68)	0.80 (0.04, 15.47)	1.89 (0.12, 29.47)	0.22 (0.01, 3.34)
**ICE Income-Race Q5 (high income, most White people)**							
**Part I: odds ratio of having any expenditure**							
Race (NH Black)	0.44 (0.27, 0.71)	0.57 (0.37, 0.89)	0.98 (0.65, 1.47)	1.52 (1.01, 2.29)	0.97 (0.54, 1.74)	0.58 (0.38, 0.89)	0.53 (0.36, 0.80)
Age	1.01 (0.99, 1.03)	1.01 (1.00, 1.03)	1.01 (0.99, 1.02)	1.01 (0.98, 1.03)	1.02 (0.98, 1.05)	1.01 (0.99, 1.02)	1.01 (1.00, 1.02)
Female	2.02 (1.26, 3.23)	1.86 (1.37, 2.52)	1.48 (1.01, 2.18)	1.07 (0.71, 1.61)	3.30 (1.69, 6.47)	1.68 (1.14, 2.47)	1.73 (1.32, 2.27)
Years of education (12)	1.57 (0.53, 4.59)	0.95 (0.32, 2.83)	1.43 (0.75, 2.72)	1.98 (0.88, 4.45)	1.09 (0.40, 2.93)	1.63 (0.85, 3.14)	2.93 (1.25, 6.89)
Years of education (>12)	2.55 (0.93, 6.93)	1.65 (0.69, 3.91)	1.76 (0.94, 3.27)	1.55 (0.73, 3.27)	0.96 (0.41, 2.28)	1.88 (0.95, 3.71)	5.5 (2.27, 13.33)
Family income level (low/middle)	0.87 (0.41, 1.84)	1.12 (0.57, 2.21)	0.82 (0.40, 1.66)	0.82 (0.41, 1.66)	0.71 (0.33, 1.47)	0.97 (0.51, 1.82)	0.57 (0.35, 0.94)
Family income (high)	0.83 (0.36, 1.90)	1.11 (0.53, 2.33)	1.18 (0.55, 2.52)	0.64 (0.33, 1.23)	0.94 (0.42, 2.08)	0.95 (0.52, 1.75)	1.10 (0.66, 1.84)
Employment status (yes)	1.28 (0.64, 2.57)	1.08 (0.61, 1.95)	1.02 (0.59, 1.78)	1.19 (0.59, 2.37)	1.09 (0.42, 2.78)	0.71 (0.38, 1.32)	0.81 (0.56, 1.15)
Insurance status (insured)	6.01 (2.52, 14.28)	6.35 (2.93, 13.75)	2.62 (0.80, 8.54)	2.14 (0.49, 9.35)	4.16 (0.49, 34.7)	3.90 (1.78, 8.54)	2.01 (0.95, 4.25)
Have a usual source of care provider (yes)	3.65 (2.14, 6.22)	3.34 (2.19, 5.08)	2.27 (1.21, 4.27)	1.03 (0.62, 1.68)	2.01 (0.84, 4.76)	2.49 (1.76, 3.53)	1.98 (1.43, 2.75)
Family having problem paying medical bills in the past 12 months (yes)	1.7 (0.83, 3.46)	1.04 (0.49, 2.22)	1.68 (1.07, 2.64)	1.9 (1.03, 3.47)	0.66 (0.27, 1.61)	2.17 (1.1, 4.26)	0.73 (0.49, 1.09)
Perceived physical health (Good-excellent)	0.26 (0.11, 0.68)	0.54 (0.31, 0.95)	1.01 (0.52, 1.96)	0.4 (0.21, 0.77)	0.20 (0.11, 0.37)	0.32 (0.15, 0.70)	0.81 (0.52, 1.26)
Perceived mental Health (Good-excellent)	0.66 (0.23, 1.82)	1.00 (0.52, 1.91)	0.78 (0.45, 1.35)	1.02 (0.59, 1.77)	0.99 (0.49, 1.99)	0.74 (0.41, 1.32)	1.15 (0.68, 1.95)
Number of comorbidities (1)	1.33 (0.83, 2.12)	1.23 (0.82, 1.86)	1.86 (0.98, 3.53)	1.66 (0.91, 3.03)	1.46 (0.51, 4.12)	2.11 (1.50, 2.97)	1.21 (0.80, 1.83)
Number of comorbidities (2-3)	5.29 (2.47, 11.32)	2.80 (1.76, 4.44)	2.27 (1.20, 4.29)	2.11 (0.97, 4.61)	1.24 (0.46, 3.37)	4.51 (2.89, 7.06)	1.38 (0.92, 2.08)

© 2023 Dean LT et al. *JAMA Health Forum.*

	Total health care expenditures	Office-based expenditures	Outpatient expenditures	Emergency room visit expenditures	Inpatient expenditures	Prescription drug expenditures	Dental care expenditures
Number of comorbidities (>=4)	13.79 (4.01, 47.36)	3.8 (1.54, 9.38)	4.00 (2.06, 7.76)	2.22 (1.06, 4.66)	1.88 (0.57, 6.16)	22.18 (11.4, 43.15)	0.91 (0.56, 1.48)
Census-tract level % with high school education	0.05 (0.01, 0.46)	0.13 (0.02, 0.85)	5.28 (0.64, 43.32)	4.59 (0.51, 41.85)	1.27 (0.07, 20.67)	0.77 (0.10, 5.71)	0.1 (0.02, 0.50)
Census-tract level % with 65+ years of age	6.12 (0.08, 420.05)	4.14 (0.18, 91.38)	0.44 (0.02, 6.58)	0.03 (0.01, 7.88)	0.05 (0.01, 2.78)	0.15 (0.01, 2.36)	0.55 (0.04, 7.35)
**Part II: cost ratios among persons with positive expenditure**							
Race (NH Black)	0.70 (0.56, 0.86)	0.75 (0.58, 0.98)	0.99 (0.55, 1.79)	0.95 (0.62, 1.43)	1.06 (0.64, 1.78)	0.77 (0.57, 1.05)	0.98 (0.70, 1.37)
Age	1.01 (1.00, 1.02)	1.00 (0.99, 1.01)	0.99 (0.97, 1.01)	0.99 (0.98, 1.01)	1.00 (0.98, 1.01)	1.00 (0.99, 1.01)	1.00 (0.99, 1.02)
Female	1.35 (1.09, 1.67)	1.21 (1.01, 1.48)	0.65 (0.37, 1.14)	0.96 (0.65, 1.43)	0.88 (0.61, 1.26)	0.93 (0.73, 1.17)	1.00 (0.79, 1.26)
Years of education (12)	1.05 (0.75, 1.47)	1.51 (0.99, 2.28)	0.90 (0.34, 2.34)	2.03 (0.74, 5.54)	0.93 (0.53, 1.63)	0.54 (0.33, 0.89)	1.87 (0.87, 4.02)
Years of education (>12)	1.24 (0.84, 1.82)	1.93 (1.24, 3.01)	0.65 (0.25, 1.67)	1.89 (0.7, 5.1)	0.92 (0.47, 1.81)	0.71 (0.41, 1.17)	1.84 (0.84, 4.04)
Family income level (low/middle)	0.83 (0.55, 1.25)	0.85 (0.55, 1.32)	1.19 (0.62, 2.26)	0.84 (0.43, 1.66)	1.50 (0.71, 3.13)	0.89 (0.55, 1.44)	0.68 (0.38, 1.21)
Family income (high)	1.21 (0.81, 1.80)	1.27 (0.88, 1.85)	1.56 (0.72, 3.40)	1.37 (0.72, 2.60)	1.59 (0.70, 3.60	1.32 (0.88, 1.97)	0.73 (0.42, 1.26)
Employment status (yes)	0.86 (0.67, 1.10)	0.82 (0.61, 1.10)	0.82 (0.47, 1.44)	1.11 (0.74, 1.65)	1.4 (0.98, 1.99)	0.77 (0.56, 1.07)	0.88 (0.6, 1.27)
Insurance status (insured)	2.15 (1.04, 4.43)	1.06 (0.32, 3.52)	9.8 (4.23, 22.72)	1.25 (0.49, 3.20)	2.98 (1.49, 5.97)	6.87 (3.73, 12.63)	0.74 (0.28, 1.95)
Have a usual source of care provider (yes)	1.35 (0.99, 1.83)	1.57 (1.16, 2.12)	0.68 (0.25, 1.85)	0.90 (0.47, 1.69)	1.03 (0.60, 1.77)	1.31 (0.77, 2.25)	0.86 (0.56, 1.33)
Family having problem paying medical bills in the past 12 months (yes)	1.39 (0.93, 2.09)	1.55 (1.02, 2.34)	3.05 (1.76, 5.26)	1.5 (0.98, 2.30)	0.87 (0.52, 1.44)	1.18 (0.8, 1.75)	1.03 (0.58, 1.83)
Perceived physical health (Good-excellent)	0.48 (0.36, 0.63)	0.68 (0.51, 0.92)	1.22 (0.55, 2.72)	0.78 (0.53, 1.15)	1.08 (0.74, 1.57)	0.44 (0.28, 0.70)	0.69 (0.45, 1.07)
Perceived mental Health (Good-excellent)	0.86 (0.62, 1.18)	0.84 (0.62, 1.13)	0.93 (0.45, 1.91)	0.74 (0.42, 1.30)	0.76 (0.48, 1.21)	0.96 (0.61, 1.51)	1.62 (1.06, 2.48)

© 2023 Dean LT et al. *JAMA Health Forum.*

	Total health care expenditures	Office-based expenditures	Outpatient expenditures	Emergency room visit expenditures	Inpatient expenditures	Prescription drug expenditures	Dental care expenditures
Number of comorbidities (1)	1.52 (1.12, 2.08)	1.87 (1.34, 2.59)	1.03 (0.41, 2.57)	0.97 (0.45, 2.10)	1.29 (0.66, 2.49)	0.64 (0.4, 1.02)	1.23 (0.78, 1.96)
Number of comorbidities (2-3)	1.51 (1.12, 2.05)	1.45 (1.06, 1.97)	1.01 (0.41, 2.42)	0.44 (0.19, 1.03)	1.41 (0.63, 3.07)	1.53 (0.96, 2.44)	1.15 (0.77, 1.72)
Number of comorbidities (>=4)	2.47 (1.71, 3.60	2.6 (1.6, 4.23)	1.17 (0.48, 2.86)	0.51 (0.25, 1.03)	1.55 (0.79, 3.02)	2.43 (1.52, 3.89)	0.93 (0.62, 1.39)
Census-tract level % with high school education	0.62 (0.20, 1.90)	0.34 (0.08, 1.41)	0.08 (0.01, 1.25)	0.5 (0.05, 4.79)	0.67 (0.11, 3.93)	1.38 (0.26, 7.11)	0.89 (0.23, 3.44)
Census-tract level % with 65+ years of age	0.36 (0.07, 1.70)	1.48 (0.24, 8.80)	0.05 (0.01, 10.9)	0.09 (0.01, 4.20)	2.94 (0.05, 147.37)	1.23 (0.14, 10.22)	0.04 (0.01, 0.28)

© 2023 Dean LT et al. *JAMA Health Forum.*
