# Supplementary material for: Health Care Expenditures for Black and White US Adults Living Under Similar Conditions
Source: JAMA Health Forum. 2023 Nov 3;4(11):e233798. doi: 10.1001/jamahealthforum.2023.3798 (PMC10625039; doi:10.1001/jamahealthforum.2023.3798)
Supplement: Supplement 2. — Data Sharing Statement [file jamahealthforum-e233798-s002.pdf]

Data Sharing Statement

Dean. Health Care Expenditures for Black and White US Adults Living Under Similar Conditions. *JAMA Health Forum*. Published November 03, 2023. doi:10.1001/jamahealthforum.2023.3798

Data
Data available: No

Additional Information
Explanation for why data not available: Data are provided from the Agency for Health Research and Quality and are not our data to share.
